# Supplementary material for: mRNA BNT162b Vaccine Elicited Higher Antibody and CD4+ T-Cell Responses than Patients with Mild COVID-19
Source: Microorganisms. 2022 Jun 18;10(6):1250. doi: 10.3390/microorganisms10061250 (PMC9228401; doi:10.3390/microorganisms10061250)
Supplement: Supplementary file 1 [file microorganisms-10-01250-s001.zip › microorganisms-1752571-supplementary.pdf]

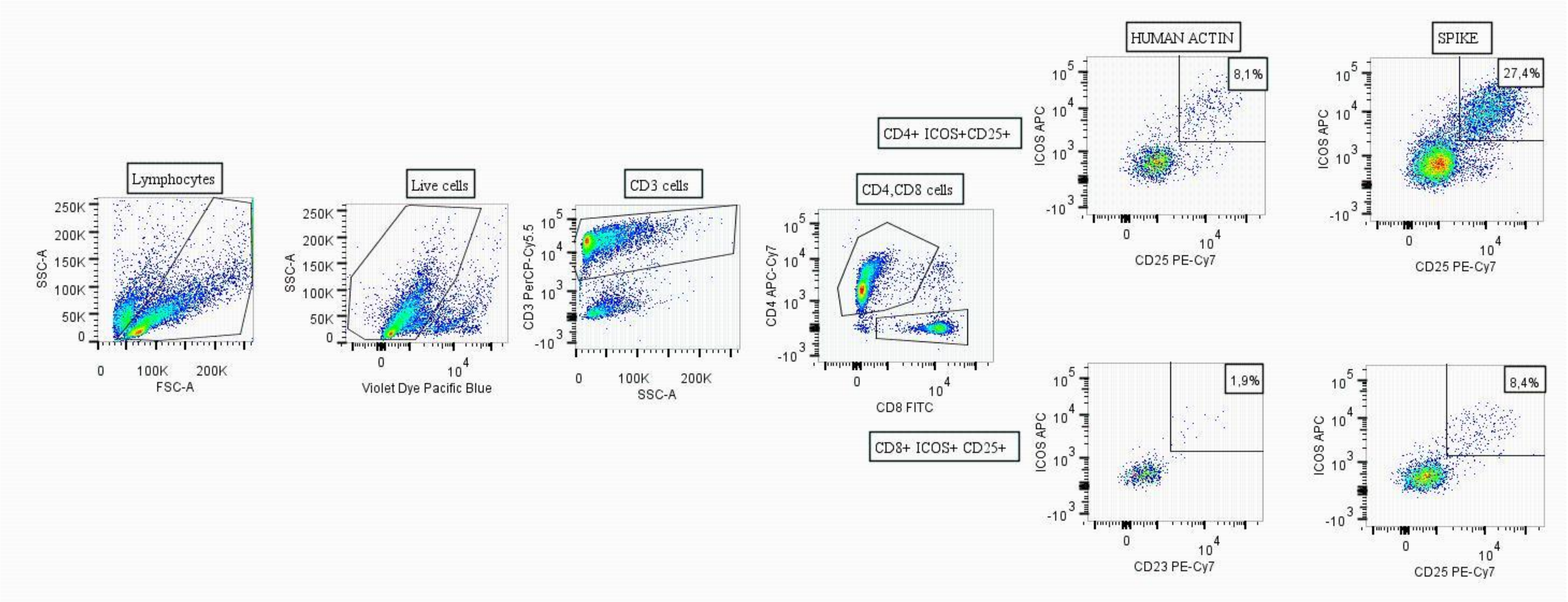


**Supplementary Figure S1.** Lymphoproliferation Phenotyping Flow Cytometry. Facs plot example on antigen-specific memory CD4+ and CD8+ T-cells from PBMC of vaccinated patient after stimulation with Spike and Human actin peptide pools. Antigen-specific T-cells were defined as CD4+ ICOS APC+ CD25 PECy7+ and CD8+ ICOS APC+ CD25 PECy7+.
